# Diurnal Changes in Distribution Characteristics of Salivary Cortisol and Immunoglobulin A Concentrations

**DOI:** 10.3390/ijerph14090987

**Published:** 2017-08-31

**Authors:** Hiromitsu Kobayashi, Chorong Song, Harumi Ikei, Bum-Jin Park, Takahide Kagawa, Yoshifumi Miyazaki

**Affiliations:** 1 Department of Nursing, Ishikawa Prefectural Nursing University, 1-1 Gakuendai, Kahoku, Ishikawa 929-1210, Japan; kobayasi@ishikawa-nu.ac.jp; 2 Center for Environment, Health and Field Sciences, Chiba University, 6-2-1 Kashiwa-no-ha, Kashiwa, Chiba 277-0882, Japan; crsong1028@chiba-u.jp (C.S.); ikei0224@ffpri.affrc.go.jp (H.I.); 3 Forestry and Forest Products Research Institute, 1 Matsunosato, Tsukuba, Ibaraki 305-8687, Japan; kagawa@ffpri.affrc.go.jp; 4 Department of Environment and Forest Resources, Chungnam National University, 99 Daehak-ro, Yuseong-gu, Daejeon 34134, Korea; bjpark@cnu.ac.kr

**Keywords:** diurnal variation, immunoglobulin A, salivary cortisol, skewness, kurtosis

## Abstract

Salivary cortisol and secretory immunoglobulin A (S-IgA) are important biomarkers for environmental and public health research. The present study investigated the diurnal variations of these salivary biomarkers, with a focus on the change of distribution characteristics (i.e., skewness and kurtosis) of the concentrations. The participants in this study were 113 healthy young males. Salivary samples were collected in the morning (6:30–7:30 a.m.) and afternoon (1:00–4:00 p.m.). The skewness and kurtosis of salivary cortisol concentrations in afternoon samples (skewness = 1.06, kurtosis = 4.78) were significantly higher than those in morning samples (skewness = 0.49, kurtosis = 2.80). The differences in skewness and kurtosis of S-IgA concentrations were non-significant; however, the standard deviation and interquartile ranges for afternoon S-IgA concentrations were significantly smaller than those for morning S-IgA concentrations. Logarithmic transformation improved the distribution of afternoon cortisol values, making it an almost normal distribution, but the transformation showed no improvement in the distribution of morning cortisol or S-IgA values. The present study explored diurnal changes in the distribution characteristics of salivary cortisol and S-IgA. Consideration of a possible diurnal change in the distribution characteristics is needed when we compare the difference in these salivary biomarkers between different times of day.

## 1. Introduction

Cortisol is one of the steroid hormones produced by the adrenal cortex. Cortisol secretion is controlled by the hypothalamic–pituitary–adrenal (HPA) axis through the secretion of corticotropin-releasing hormone and adrenocorticotropin. The HPA axis is the central component of stress response systems, and cortisol concentrations have been used as a biological stress indicator [1]. Although free cortisol can be detected in serum, urine, hair, and saliva, analysis of salivary cortisol concentrations is noninvasive and thus advantageous [2]. In fact, salivary cortisol concentrations have been used as a noninvasive stress marker in various biomedical studies, such as job strain [3], school stress [4,5], noise [6,7], or maltreatment of children [8,9]. Salivary cortisol measurement is also used to evaluate the restorative effect of natural environments [10,11]. In addition to cardiovascular measurements (heart rate, blood pressure), salivary cortisol concentration is one of the most frequently used variables in studies on the effect of natural environments [12].

Cortisol exhibits a large diurnal variation. Salivary cortisol concentrations increase in the morning, peaking at approximately 30 min after awakening in the morning and gradually decreasing throughout the day. The magnitude of this phenomenon—known as the cortisol awakening response (CAR) [13,14]—has recently garnered attention as an HPA activity indicator [15,16]. It has been suggested that a blunted CAR is associated with psychosocial disorders [17] and poor health outcomes [17,18].

An indicator of immune function is a useful biomarker for environmental and public health research [19] as well as salivary cortisol measurement. Secretory immunoglobulin A (S-IgA) is the most abundant immunoglobulin in saliva, and plays a critical role in mucosal immunity [20]. S-IgA concentrations were used as a noninvasive indicator of immunity [21] and/or environmental stress [22]. Several studies reported that salivary S-IgA concentrations also exhibited diurnal variations. Importantly, S-IgA and salivary cortisol concentrations show different patterns of diurnal variations, especially in the morning. Unlike salivary cortisol levels, S-IgA concentrations peak immediately after awakening and decline steadily afterwards [23,24]. Despite this difference in the pattern of change in the morning, the concentrations of both salivary biomarkers are generally higher in the morning and lower in the afternoon [25,26]. 

Diurnal variations of both salivary cortisol and S-IgA concentrations were investigated, with a focus on variations in mean concentrations. Mean concentration is a significant albeit merely representative value within a target population. Conversely, studies focusing on individual variations might be challenging, as they require larger sample sizes; thus, most studies so far have focused on mean values of physiological functions [27], and reports on investigations of the distribution characteristics of individual variations are limited. The present study aimed to determine diurnal variations of salivary cortisol and S-IgA concentrations in 113 young Japanese males to provide a new perspective on the diurnal variations of both salivary biomarkers.

## 2. Materials and Methods

### 2.1. Participants

A total of 113 Japanese male students participated in the present study. Demographic characteristics of all subjects are shown in Table 1. None of the participants reported a history of physical or psychiatric disorders. During the study period, alcohol and tobacco consumption was prohibited, and caffeine consumption was controlled. The study was conducted under the regulations of the Institutional Ethical Committee of the Forestry and Forest Products Research Institute, Japan (project identification code number: 16-558).

### 2.2. Experimental Procedures and Salivary Cortisol Measurement

Salivary samples were collected two times (morning and afternoon) on the day of experiment from each participant. Morning samples were collected before breakfast and teeth brushing, approximately 20–40 min after awakening (6:30–7:30 a.m.). Afternoon samples were collected after lunch. Although the time of saliva collection in the afternoon varied from 1:00 to 4:00 p.m., more than 90% of the samples were collected between 1:30 and 3:30 p.m.

Salivary samples were collected from each participant using a Salivette^®^ (Sarstedt, Nümbrecht, Germany). All samples were immediately frozen and transported to the laboratories of SRL, Inc. (Tokyo, Japan). Each sample consisted of one 0.25-mL aliquot of saliva; cortisol concentrations were analyzed by radioimmunoassay, whereas S-IgA concentrations were analyzed by enzyme immunoassay.

### 2.3. Statistical Analysis

Mean, median, standard deviation (SD), coefficient of variation (CV), quartile 1 (Q1; 25 percentile), quartile 3 (Q3; 75 percentile), interquartile range (IQR; Q3−Q1), skewness, and kurtosis of distribution were calculated for both salivary cortisol and S-IgA concentrations. Differences between morning and afternoon values for these parameters were tested by a permutation test. A permutation test is a nonparametric statistical test that uses a resampling method. In this study, resampling was performed 5000 times. *p*-values were calculated according to a method proposed by Phipson and Smyth [28]. A *p*-value < 0.05 was considered statistically significant for the permutation test. An uncertainty of the *p*-value around 0.05 was estimated as 0.3%.

The normality of the distribution curve of salivary biomarkers was tested by the Jarque–Bera (JB) test [29]. A larger JB value implies that the samples are further from a normal distribution. If data are sampled from a normal distribution, JB statistics follow a chi-squared distribution with two degrees of freedom. Similar to the permutation test, a *p*-value < 0.05 was considered statistically significant for the JB test.

Similar analyses were performed on natural logarithm-transformed concentrations of the biomarkers. Statistical tests of log-transformed concentrations for Q1, Q3, and IQR were not performed because the statistical tests for these values produced results identical to those of the analyses of raw concentrations.

## 3. Results

**Figure 1:**
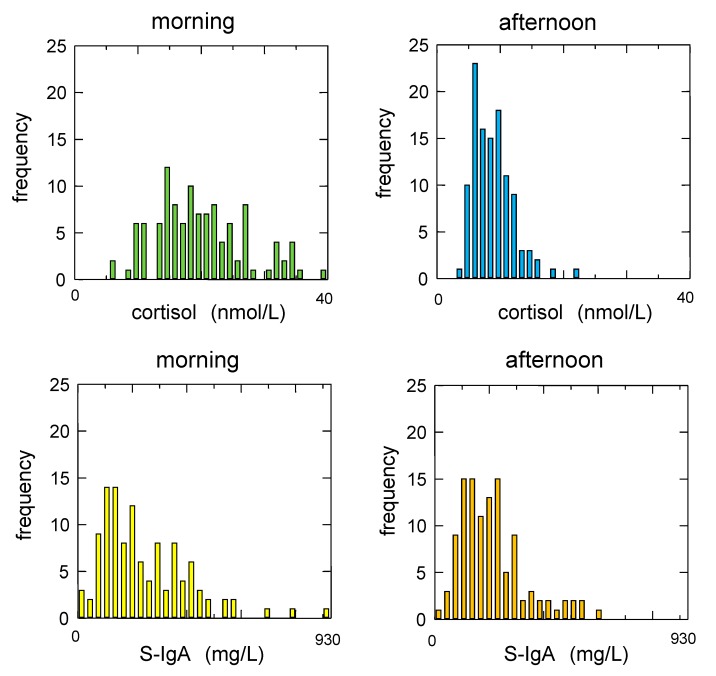
Frequency distributions of salivary cortisol and immunoglobulin A concentrations. Salivary cortisol concentrations showed a more skewed distribution in the afternoon compared to those in the morning (upper panels), whereas the skewness of secretory immunoglobulin A (S-IgA) concentrations did not change from morning to afternoon (lower panels).

The distribution characteristics of salivary cortisol and S-IgA concentrations are summarized in Table 2, and the frequency distributions of salivary biomarkers plotted using histograms are presented in Figure 1.

Morning cortisol concentrations (mean, 19.97 nmol/L; median, 18.76 nmol/L) were markedly higher than those in the afternoon (mean, 8.84 nmol/L; median, 8.28 nmol/L); the permutation test revealed that the differences in both means and medians were statistically significant (*p* < 0.001). SD, Q1, Q3, and IQR values were significantly smaller for the afternoon cortisol samples than the morning samples. However, CV did not differ between the morning and afternoon samples, as the decrease in SD was proportional to the mean. 

The skewness of morning and afternoon cortisol concentrations were 0.49 and 1.06, respectively; a more skewed distribution curve was observed in afternoon cortisol levels. The difference in skewness between morning and afternoon cortisol concentrations was statistically significant (*p* = 0.022). Kurtosis of the distribution also significantly increased from morning to afternoon (2.80 vs. 4.78, *p* = 0.008). According to the JB test, the distribution curve of morning salivary cortisol concentrations was not significantly different from a normal distribution.

Similar to that observed for salivary cortisol concentrations, there was a decrease in mean S-IgA concentrations from morning (245.0 mg/L) to afternoon (211.3 mg/L); however, this decline was not significant (*p* = 0.078). Compared with the change observed in mean S-IgA, the change in median S-IgA was unclear (*p* = 0.504).

The distribution of afternoon S-IgA concentrations demonstrated smaller SD and IQR than that of the morning S-IgA values. In contrast to that observed for cortisol, the decrease in SD of S-IgA was not proportional to the decrease in mean S-IgA. Thus, there was a decrease in CV, although this was not statistically significant. Evaluation of quartile values showed that Q3 in afternoon S-IgA concentrations was significantly smaller than that in morning values (265.7 mg/L vs. 349.4 mg/L, *p* = 0.021), whereas Q1 was not different between morning and afternoon concentrations (123.5 and 123.7 mg/L, respectively). 

Skewness of the S-IgA distribution between morning and afternoon concentrations was nearly unchanged (1.34 and 1.12, respectively). Although kurtosis of the S-IgA distribution was larger in the morning than in the afternoon (5.46 vs. 4.12), the difference was also nonsignificant. According to the JB test, the distribution curves of both morning and afternoon S-IgA concentrations were determined to be significantly different from a normal distribution; however, the JS statistic was larger in morning S-IgA concentrations. 

The distribution characteristics of log-transformed salivary cortisol and S-IgA concentrations are summarized in Table 3. Significant differences were observed between morning and afternoon log-transformed cortisol concentrations in terms of mean and median. Log-transformed morning cortisol concentrations exhibited a slightly left-skewed and peaked distribution (skewness = −0.51; kurtosis = 3.40; JB = 5.7). Skewness and kurtosis for log-transformed afternoon cortisol concentrations were 0.10 and 2.66, respectively. A smaller JB value (0.7) was observed in log-transformed afternoon cortisol concentrations compared with the value for the raw concentration (36.1). Log transformation morning and afternoon S-IgA concentrations produced a left-skewed and peaked distribution. In particular, a markedly larger kurtosis (12.57) and JB value (495.0) were observed in the distribution of afternoon S-IgA concentrations.

## 4. Discussion

### 4.1. Diurnal Changes in Salivary Cortisol

In the present study, we found that mean afternoon salivary cortisol concentration was lower than that in the morning. Smyth et al. [30] reported a decline in mean salivary cortisol concentration from approximately 16 nmol/L at 8:00 a.m. to 7 nmol/L at 2:00 p.m. A similar decline from approximately 16 nmol/L at 9:00 a.m. to 6–8 nmol/L at 2:00–3:00 p.m. was also demonstrated by Kirschbaum et al. [31]. The cortisol concentrations in the present study were slightly higher than those reported previously; however, the rate of decline in salivary cortisol concentrations was nearly identical. 

The present study investigated alterations in the distribution curve as well as changes in representative values (i.e., the mean and median). SD of salivary cortisol levels also decreased in proportion to the decrease in its mean value; thus, CV did not change. Our analysis with the JB test revealed that the variation of afternoon cortisol concentrations was significantly different from a normal distribution, whereas the variation of morning cortisol concentrations was not. Given that the skewness and kurtosis of a normal distribution are 0 and 3, respectively, in the current study, these values for morning salivary cortisol concentrations were considered to reflect a normal distribution.

In a previous study investigating the distribution characteristics of morning salivary cortisol concentrations [32], we found that the skewness of the morning salivary cortisol concentrations was considerably smaller than that of the afternoon salivary cortisol concentrations reported by other researchers [33,34]. Therefore, we hypothesized that diurnal alterations in the distribution curve of salivary cortisol concentrations accompanied changes in mean concentrations, and this was validated in the present study, which demonstrated that salivary cortisol concentrations showed significantly larger skewness in the afternoon than in the morning. The skewed distribution in afternoon salivary cortisol concentrations might be the result of a floor effect [35], which might have prohibited negative values when salivary cortisol concentrations were lower in the afternoon, resulting in a distorted distribution with a shorter left tail (positive skewness).

### 4.2. Diurnal Changes in Secretory Immunoglobulin A

Several researchers reported a marked decline in S-IgA concentrations throughout the day. For example, two studies found that the difference between the minimum and maximum for one day was twofold [26] or fourfold [25]. Conversely, others reported a small and/or insignificant diurnal variation in S-IgA [23,24]. While we found that the diurnal variation in mean S-IgA concentrations was inconclusive, our finding was in agreement with some of the previous studies.

Differences in skewness and kurtosis of S-IgA distribution were also insignificant, although a lower kurtosis (a more flattened curve) was observed in afternoon S-IgA concentrations. Assessment by the JB test indicated that the variation of S-IgA concentrations was significantly different from a normal distribution in both the morning and the afternoon. However, the distortion from normal distribution was determined to be larger in the morning than in the afternoon, as the JB value was larger in the morning.

Assessment of variations in S-IgA concentrations revealed that the SD and IQR of S-IgA concentrations were significantly smaller in the afternoon than in the morning, implying that the inter-individual variation of S-IgA concentrations decreased from morning to afternoon.

The Q3 value of S-IgA concentrations was significantly decreased from morning to afternoon, whereas Q1 remained nearly unchanged from morning to afternoon. From these results, decreased inter-individual variation of S-IgA concentration might be attributable to the decrease in Q3 and not to the decrease in Q1. These asymmetrical changes in Q1 and Q3 could lead to a change in skewness and/or kurtosis of the distribution; however, diurnal changes in the skewness and kurtosis of S-IgA were not significant in the present study. This result might be due to the smaller sample size and lower statistical power to test differences in higher moment statistics. Larger sample size and comparisons over a longer time span—such as a comparison between early morning and late night—might more clearly reflect potential changes in skewness and/or kurtosis for S-IgA concentrations, similar to those observed for salivary cortisol concentrations.

### 4.3. Log Transformation of Salivary Cortisol and Secretory Immunoglobulin A Concentrations

In addition to raw concentrations, log-transformed cortisol and S-IgA concentrations were also analyzed. Log-transformed afternoon cortisol concentrations exhibited a small JB value (0.7), implying that the distribution was almost normal. In contrast, log transformation of the morning cortisol values produced negative skewness and a slightly larger JB value (5.7) compared with that of raw cortisol values (4.7). These results suggested that log transformation of morning cortisol values has an excessive effect, although the transformation of afternoon cortisol values is effective for improving the distribution. In many previous studies, log transformation has been applied to cortisol concentrations for statistical processing (e.g., Adam and Kumari [18], Gordis et al. [34], Hansen et al. [36], Beli and Hanes [37], Turner-Cobb et al. [38], Sephton et al. [39], Yehuda et al. [40]). However, log transformation should be applied only to afternoon cortisol concentrations. When log transformation is applied to early morning cortisol concentrations, the transformation may exacerbate the distribution characteristics.

The effect of log transformation on the distribution of S-IgA values was also examined. The transformation produced negative skewness in both morning and afternoon S-IgA concentrations. According to the results of JB tests, the distributions of the transformed S-IgA values were more distorted than the distribution of raw S-IgA values. In some studies, log transformation has also been applied to S-IgA values (e.g., Phillips et al. [41], Moreira et al. [42], Laurent et al. [43]). Considering the results of this study, log transformation is inappropriate for the statistical analysis of S-IgA values.

## 5. Conclusions

It has been considered that salivary cortisol concentration exhibits skewed and/or kurtotic distribution. This study confirmed the non-normal distribution in salivary cortisol concentration measured in the afternoon. However, approximately normal distribution was demonstrated in salivary cortisol measured in the morning. Regarding S-IgA concentration, smaller inter-individual variation can be expected in the afternoon compared with in the morning, although the change of mean S-IgA was small and insignificant. Logarithmic transformations have been applied on cortisol concentration for statistical analysis in many studies. However, log transformation of morning cortisol and S-IgA values has no effect or produced a more distorted distribution, although it was effective for afternoon cortisol values. A non-parametric test or square-root transformation might be more appropriate than log transformation for analyzing these salivary biomarkers.

In conclusion, diurnal changes in the distribution characteristics of these salivary biomarkers were explored in this study. Consideration of a possible diurnal change in the distribution characteristics is needed when we compare a difference in salivary cortisol or S-IgA between different times of day.

## Figures and Tables

**Table 1:** Demographic parameters of the participants (n = 113).

Variable	Age (Years)	Height (cm)	Weight (kg)
Mean	22.3	172.0	65.6
SD *	1.6	5.7	10.2
Max	29	187.5	110.0
Min	20	155.0	50.0

* SD: standard deviation.

**Table 2:** Characteristics of the distribution of salivary cortisol and immunoglobulin A concentrations.

Variable	Cortisol (nmol/L)			S-IgA (mg/L)		
	Morning	Afternoon	*p*	Morning	Afternoon	*p*
Mean	19.97	8.84	<0.01 **	245.0	211.3	0.08
Median	18.76	8.28	<0.01 **	204.0	189.1	0.50
SD	7.17	3.12	<0.01 **	163.7	119.4	0.03 *
CV	35.9%	35.3%	0.90	66.8%	56.5%	0.17
Q1	15.17	6.62	<0.01 **	123.5	123.7	0.94
Q3	24.00	10.48	<0.01 **	349.4	265.7	0.02 *
IQR	8.83	3.86	0.01 *	225.9	142.0	0.01 *
Skewness	0.49	1.06	0.02 *	1.34	1.12	0.80
Kurtosis	2.80	4.78	<0.01 **	5.46	4.12	0.80
JB	4.7	36.1		62.3	29.5	
	*p* = 0.10	*p* < 0.01 **		*p* < 0.01 **	*p* < 0.01 **	

S-IgA, secretory immunoglobulin A; SD, standard deviation; CV, coefficient of variation (SD/mean); Q1, quartile 1 (25 percentile); Q3, quartile 3 (75 percentile); IQR, interquartile range (Q3−Q1); skewness, parameter of symmetry; kurtosis, parameter of peak (positive) or flat (negative) distribution. Skewness and kurtosis of a normal distribution are 0 and 3, respectively; JB, Jarque–Bera value. A larger JB value implies that the samples are further from a normal distribution; * *p* < 0.05 and ** *p* < 0.01.

**Table 3:** Characteristics of the distribution of log-transformed salivary cortisol and immunoglobulin A concentrations.

Variable	Cortisol (ln(nmol/L))			S-IgA (ln(mg/L))		
	Morning	Afternoon	*p*	Morning	Afternoon	*p*
Mean	2.93	2.12	<0.01 **	5.26	5.18	0.44
Median	2.93	2.11	<0.01 **	5.32	5.24	0.50
SD	0.38	0.34	0.29	0.78	0.66	0.42
CV	13.0%	16.1%	0.06	14.9%	12.85%	0.48
Skewness	−0.51	0.10	<0.01 **	−1.15	−1.84	0.61
Kurtosis	3.40	2.66	<0.01 **	6.01	12.57	0.09
JB	5.7	0.7		67.6	495.0	
	*p* = 0.06	*p* = 0.70		*p* < 0.01 **	*p* < 0.01 **	

S-IgA, secretory immunoglobulin A; SD, standard deviation; CV, coefficient of variation (SD/mean); skewness, parameter of symmetry; kurtosis, parameter of peak (positive) or flat (negative) distribution. Skewness and kurtosis of a normal distribution are 0 and 3, respectively; JB, Jarque–Bera value. A larger JB value implies that the samples are further from a normal distribution; * *p* < 0.05 and ** *p* < 0.01.
